# Low-Dose Computed Tomography Scanning Protocols for Online Adaptive Proton Therapy of Head-and-Neck Cancers

**DOI:** 10.3390/cancers14205155

**Published:** 2022-10-21

**Authors:** Konrad P. Nesteruk, Mislav Bobić, Gregory C. Sharp, Arthur Lalonde, Brian A. Winey, Lena Nenoff, Antony J. Lomax, Harald Paganetti

**Affiliations:** 1 Department of Radiation Oncology, Massachusetts General Hospital and Harvard Medical School, Boston, MA 02114, USA; 2 Department of Physics, ETH Zurich, CH-8093 Zurich, Switzerland; 3 Center for Proton Therapy, Paul Scherrer Institute, CH-5232 Villigen, Switzerland

**Keywords:** adaptive proton therapy, low-dose CT, CT-on-rails, Monte Carlo, head-and-neck cancers

## Abstract

**Simple Summary:**

Adaptive proton therapy requires taking images over the course of treatment to correct the plan for anatomy changes. Most workflows assume daily imaging for this purpose. The associated imaging doses can be significant, which may compromise one of the proton therapy aims: minimizing the dose to healthy tissue. Low-dose scanning protocols address this problem. In this paper, we evaluate the influence of low-dose CT protocols on adaptation. We used a head phantom to define the protocols and simulated adaptive treatments of 10 head-and-neck patients with our established adaptation framework. We assessed the influence of lower image quality on the contour propagation and dose calculation. We demonstrated that decreasing the imaging dose by a factor of 40 with respect to our standard CT scanning protocol does not affect the adaptation performance.

**Abstract:**

Purpose: To evaluate the suitability of low-dose CT protocols for online plan adaptation of head-and-neck patients. Methods: We acquired CT scans of a head phantom with protocols corresponding to CT dose index volume CTDI_vol_ in the range of 4.2–165.9 mGy. The highest value corresponds to the standard protocol used for CT simulations of 10 head-and-neck patients included in the study. The minimum value corresponds to the lowest achievable tube current of the GE Discovery RT scanner used for the study. For each patient and each low-dose protocol, the noise relative to the standard protocol, derived from phantom images, was applied to a virtual CT (vCT). The vCT was obtained from a daily CBCT scan corresponding to the fraction with the largest anatomical changes. We ran an established adaptive workflow twice for each low-dose protocol using a high-quality daily vCT and the corresponding low-dose synthetic vCT. For a relative comparison of the adaptation efficacy, two adapted plans were recalculated in the high-quality vCT and evaluated with the contours obtained through deformable registration of the planning CT. We also evaluated the accuracy of dose calculation in low-dose CT volumes using the standard CT protocol as reference. Results: The maximum differences in *D*_98_ between low-dose protocols and the standard protocol for the high-risk and low-risk CTV were found to be 0.6% and 0.3%, respectively. The difference in OAR sparing was up to 3%. The Dice similarity coefficient between propagated contours obtained with low-dose and standard protocols was above 0.982. The mean 2%/2 mm gamma pass rate for the lowest-dose image, using the standard protocol as reference, was found to be 99.99%. Conclusion: The differences between low-dose protocols and the standard scanning protocol were marginal. Thus, low-dose CT protocols are suitable for online adaptive proton therapy of head-and-neck cancers. As such, considering scanning protocols used in our clinic, the imaging dose associated with online adaption of head-and-neck cancers treated with protons can be reduced by a factor of 40.

## 1. Introduction

Adaptive approaches in radiotherapy consider geometrical changes in the patient to ensure delivery of the prescribed dose to the target while sparing the healthy tissues according to predefined constraints. The geometrical changes refer to patient setup and interfraction anatomy variations. The latter can be due to the patient’s weight loss, change in tumor volume, or even sinus filling. Adaptive approaches can be particularly beneficial for proton therapy, where the daily variations in a patient’s geometry can affect the range resulting in cold spots in the target or hot spots in organs at risk (OARs). As summarized in [1], there are several proton adaptation workflows published [2,3,4,5,6,7,8,9,10,11], including one developed at the Massachusetts General Hospital (MGH) [8,9]. Daily imaging required for adaptive treatments is associated with significant imaging doses and may compromise one of proton therapy aims to minimize the dose to healthy tissue. Moreover, there are general concerns about the overuse of computed tomography (CT) [12,13]. Cone-beam CT (CBCT) also leads to high extra doses, which are comparable to fan-beam CT or might be even higher [14]. Therefore, a significant reduction in the imaging doses without compromising the dosimetric efficacy of adaptation would make on-line adaptive approaches much more attractive. The imaging dose is proportional to the product of tube current and exposure time, commonly known as mAs. Therefore, the dose can be reduced by decreasing the tube current, exposure time, or both. This, however, leads to a lower quality of the resulting image. It may affect the accuracy of dose calculation and, in the case of some workflows, the accuracy of contour propagation from the planning CT to the image representing the anatomy of the day. Most adaptive proton workflows published assume the use of CT for daily imaging. The workflow developed at MGH was initially conceived for CBCT, but we also considered its application in centers equipped with CT-on-rails [15]. One of the most important advantages of using fan-beam CT is the much better image quality. Therefore, low-dose protocols might be sufficient for effective adaptation. In fact, the adaptive workflow established at the Paul Scherrer Institute (PSI, Switzerland), which involves daily imaging with in-room CT-on-rails, assumes the use of low-dose CT protocols [16]. A group from KU Leuven in Belgium developed a low-dose CT simulation to optimize CT acquisition protocols to decrease the radiation dose to the patients [17]. Their primary motivation is to find the lowest possible CT radiation dose that still guarantees sufficient information for proton therapy treatment planning. Using the developed tool, they have conducted a study on the accuracy of the dose calculation and plan optimization in proton therapy based on low-dose CT [18].

Despite the interest in using low-dose CT in proton therapy and adaptive approaches, in particular, there is no quantitative study that shows if a clinically available CT scanner might be used for daily imaging with significantly reduced imaging doses to the patient without compromising adaptation efficacy. Therefore, the goal of our work is to test how the decreased quality of a daily CT image would affect adaptive proton treatments of head-and-neck (H&N) cancers. Since our adaptive workflow involves contour propagation by means of deformable image registration and Monte Carlo dose calculations, this study covers both potential uncertainties associated with lower image quality: contour propagation and dose calculation. 

## 2. Materials and Methods

### 2.1. Patient Cohort and Treatment Planning

The patient cohort and treatment plans used for this study were identical to those used in our previous works [8,15,19]. The dataset includes 10 H&N patients with the planning CT image and daily acquired CBCT images. In this study, for each patient, we chose from 31–35 fractions only the fraction with the largest patient geometry changes. Degradation of the non-adapted base plan calculated on such a fraction was the largest, as quantified by DVH metrics. For each CBCT image, a virtual CT (vCT) was created using deformable image registration (DIR) by deforming the original planning CT to the CBCT image, as described in [15]. Therefore, the quality of each vCT was consistent with the standard imaging protocol used in our clinic (tube current 400 mA, CTDI*_vol_* = 164 mGy). 

All base plans were designed as 57 Gy(RBE) and 70 Gy(RBE) prescribed to low-risk and high-risk CTV, respectively. Since online adaptation is expected to allow for a significant reduction in the margins and the study focuses on relative comparisons, the plans do not include any PTV or range uncertainty margins representing the idealistic best-case scenario. For both CTVs, the clinical objectives were defined as *D*_98_ ≥ 95% and *D*_2_ ≤ 107% of the prescribed dose, where *D*_98_ and *D*_2_ are the minimum doses to 98% and 2% of the CTV volume, respectively. We considered the following organs at risk (OARs) with the corresponding constraints: spinal cord (*D_max_* < 45 Gy), parotid glands (*D_mean_* < 26 Gy), constrictor muscles (*D_mean_* < 42 Gy), larynx (*D_mean_* < 40 Gy), and brainstem (*D_max_* < 54 Gy). 

The plans were created in Ray Station with three fields (60°, 180°, and 300°), each using a range shifter with a water equivalent thickness of 40 mm and a 30 mm minimum air gap. The *IBA Dedicated Nozzle* beam model was used for planning and dose calculations with the spot sigma in air ranging from 2.5 to 6.4 mm for the applicable nominal beam energies (between 225 MeV and 65 MeV, respectively). 

### 2.2. Phantom Measurements and Low-Dose CT Scanning Protocols

**Figure 1:**
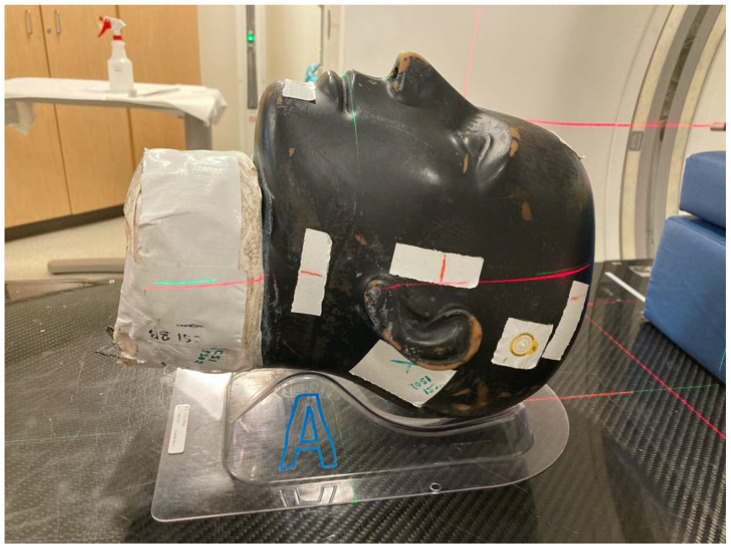
Head phantom used to establish low-dose scanning protocols.

We scanned a head phantom using a GE Discovery RT scanner (Figure 1). This scanner is routinely used in our clinic for CT simulations of radiation treatments. We defined 7 scanning protocols corresponding to different settings of the tube current. The scanning parameters used for phantom measurements are reported in Table 1. The highest tube current (400 mA) is almost identical to the standard scanning protocol used in the clinic (395 mA). The lowest tube current (10 mA) is the minimum achievable setting in this particular scanner. Therefore, our protocols correspond to the volume CT dose index (CTDI*_vol_*) in the range 4.2–166 mGy. The CTDI*_vol_* was measured on a 16 cm head phantom. For image reconstruction, we used the filtered back projection algorithm, routinely employed in the clinic. The position of the phantom was not changed between consecutive scans to ensure the alignment of the obtained images. 

**Figure 2:**
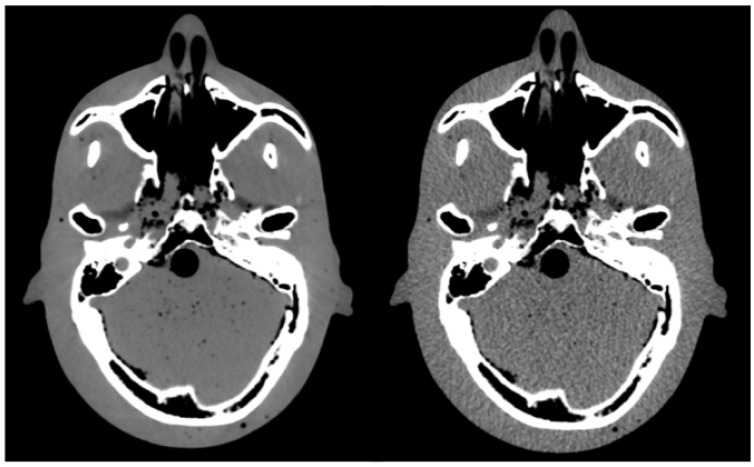
Pair of CT images of head phantom: standard protocol (**left**) and low-dose protocol (**right**).

**Figure 3:**
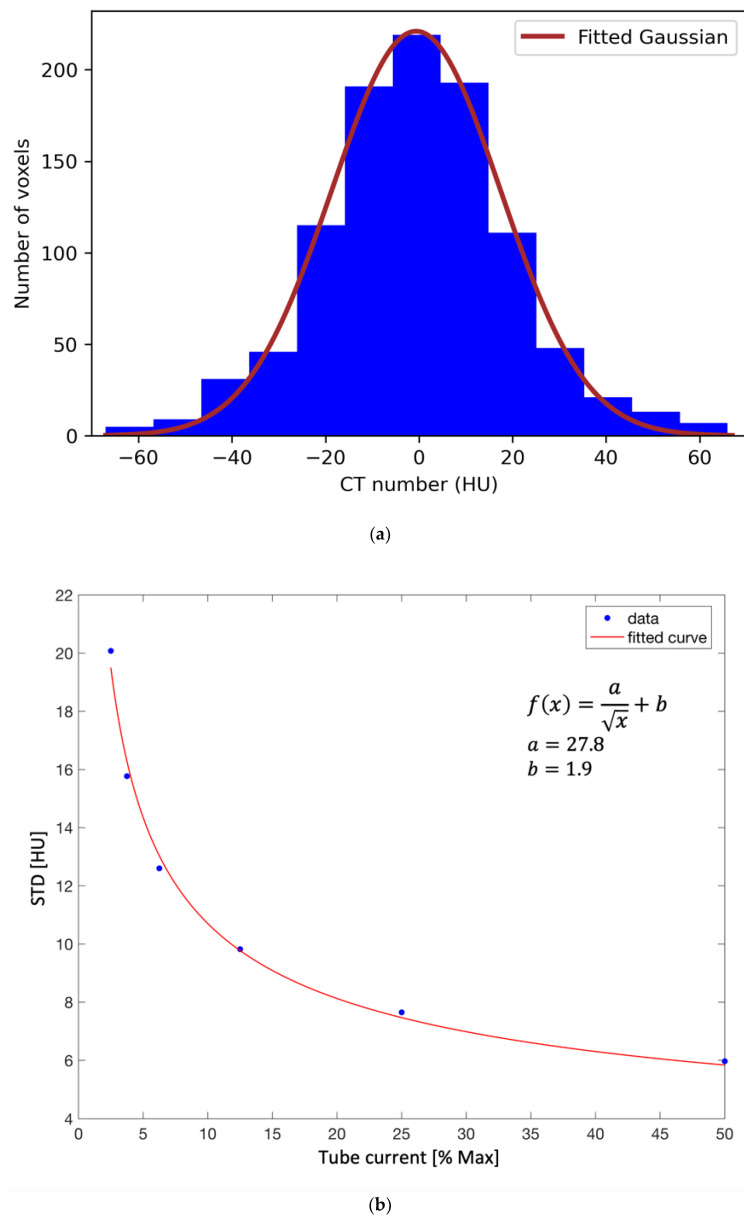
(**a**) Distribution of CT numbers in the lowest-dose CT image of the head phantom corresponding to a CT number of 0 HU in the standard-protocol CT image. A Gaussian function is fitted to the histogram. (**b**) Standard deviation (STD) for a CT number of 0 HU as a function of the tube current.

A pair of images (standard-protocol and corresponding low-dose) were analyzed for each of the six low-dose scanning protocols, as shown in Figure 2 for the lowest-dose protocol (CTDI*_vol_* = 4.2 mGy). The images were analyzed using SimpleITK—an open source interface to the image analysis toolkit Insight Toolkit (ITK) [20,21,22,23]. Due to the increased noise, each CT number in the standard-protocol CT image was represented by a distribution of CT numbers in the corresponding low-dose CT image. Therefore, voxels with the same CT number in the standard-protocol CT image had a range of different HU values in the low-dose CT image. We analyzed all the voxels in 11 central sagittal slices (the central slice corresponding to the middle of the phantom and 5 slices in each lateral direction from the central slice) so that most CT numbers occurring in the standard CT image were included in the analysis while optimizing the computation time. An example is shown in Figure 3a for the lowest-dose protocol, where a CT number of 0 HU in the standard-protocol CT image corresponds to a distribution with a mean value of −0.45 HU and a standard deviation of 20 HU. Based on the obtained distributions, the noise can be assumed Gaussian, which is demonstrated in Figure 3a with the best fit of a Gaussian function to the histogram (least-square method). For each CT number in the reference standard-protocol CT, we calculated mean value and standard deviation of the CT numbers in the corresponding voxels of the low-dose image. A lookup table that mapped original CT numbers to the calculated mean and standard deviation of the corresponding distribution was composed. Calculating the mean and standard deviation of the sample to estimate the parameters μ and σ of the distribution was found more reliable than fitting a Gaussian function to each histogram. Least-square fitting is sensitive to histogram binning, which would have to be carefully chosen for each distribution, as the number of entries in the histogram depends on how frequently a given CT number occurs in the reference image. Figure 3b shows the dependency of the standard deviation on the tube current for a CT number of 0 HU. The tube current is expressed as the percentage of the maximum tube current of 400 mA (standard protocol). The dependency is consistent with the Poisson distribution, as the number of photons is proportional to the tube current, and the lower number of events, the higher uncertainty on the CT number. 

**Figure 4:**
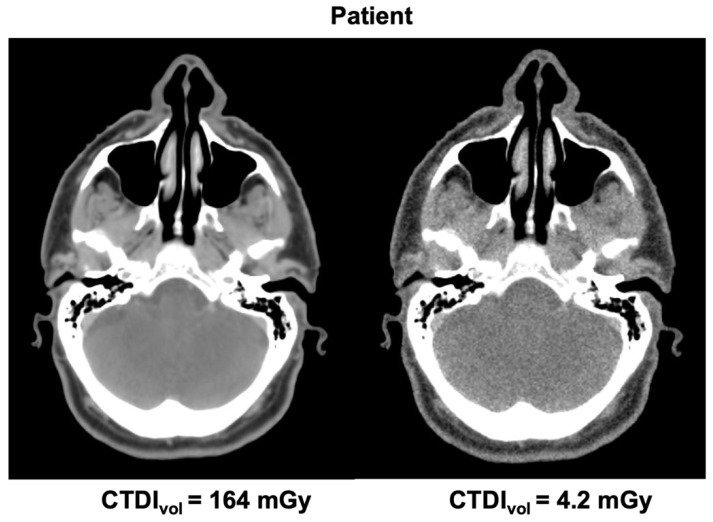
Pair of vCT images of a patient: standard protocol (**left**) and low-dose protocol (**right**).

In order to produce synthetic low-dose vCT images for each patient, each voxel in the original vCT was assigned with a CT number randomly chosen from a Gaussian distribution with the mean and sigma taken from the lookup table. Since only a subset of voxels in phantom images were analyzed to limit the computing time (11 central sagittal slices), we performed quality assurance of the synthetic low-dose vCTs with the criterion of at least 99.9% voxels being assigned with a new CT number. Figure 4 shows a pair of vCT images for one patient: original (standard protocol) and synthetic corresponding to the lowest dose (CTDI*_vol_* = 4.2 mGy).

### 2.3. Influence of Low-Dose Scanning Protocols on Online Adaptive Treatments

Our framework for online adaptive proton therapy [8] is based on fast GPU–accelerated Monte Carlo (GPU-MC) calculations. For this study, the *gPMC* code was used [24,25,26,27], while the current version of the adaptive framework is based on a newly developed GPU-MC code with an efficient data structure: *Moqui* [28]. Contour propagation is realized by applying DIR of the planning CT to daily image, which for this study was vCT. We applied DIR using the B-spline algorithm with the mean squared error metric in Plastimatch—an open-source code for radiotherapy and imaging [29,30]. The adaptation is performed by calculating the dose with a new set of contours and adjusting the weights of the beamlets based on the dose-influence matrix [9,31]. Adapted plans are then verified by calculating the dose in the image volume and scoring the dose for the propagated contours.

**Figure 5:**
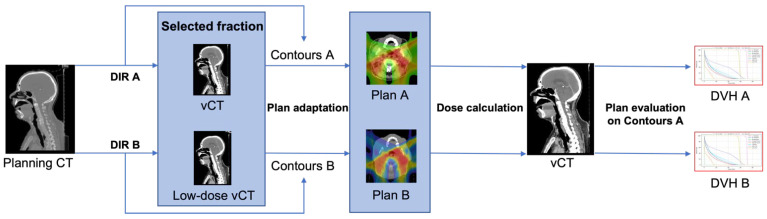
Overview of the methodology to assess the influence of CT scanning protocols on adaptive treatments.

As previously mentioned, low-dose CT scanning protocols may affect online adaptation by increasing DIR and dose calculation uncertainties. In order to test the actual impact of both uncertainties, we simulated one fraction of the adaptive treatment for 10 H&N patients using different CT scanning protocols, as shown in Figure 5. Each low-dose protocol (low-dose vCT) was compared with the reference standard protocol (vCT). By registering planning CT to the vCT and low-dose vCT (DIR A and DIR B, respectively), a set of contours was obtained (Contours A and Contours B, respectively). Contours A and Contours B were used to optimize the base plan, and adjusted Plan A and Plan B were obtained, respectively. The dose was calculated on Contours A for both vCT and low-dose vCT to see the potential influence of using low-dose CT on the resulting target coverage and OAR sparing with respect to the adaptation based on the standard-protocol CT (DVH A and DVH B). 

We calculated standard DVH metrics for each pair: the reference standard protocol and a low-dose protocol represented by DVH A and DVH B, respectively. For the target coverage, we used *D_98_* for high-risk and low-risk CTVs. For organs at risk, we evaluated the mean dose for the larynx, parotids, constrictors, and *D_1cc_* (the minimum dose to the most irradiated 1 cc) for the spinal cord. Then, for each low-dose protocol studied, we calculated the percentage difference with respect to the reference standard protocol.

In order to assess the influence of using low-dose CT scans on contour propagation, we calculated the Dice similarity coefficient [32] between contours (Contours A and Contours B in Figure 5) for each low-dose protocol. We considered three different contours: high-risk CTV, low-risk CTV, and a union of all OARs.

Eventually, we evaluated the accuracy of Monte Carlo dose calculation using low-dose CT volumes with respect to the standard-protocol CT volumes. For this purpose, we chose the lowest-dose protocol (CTDI*_vol_* = 4.2 mGy) corresponding to the worst-case scenario and a non-adapted base plan. For 6 patients, the dose was calculated using a 1.0 mm × 1.0 mm × 2.5 mm dose grid, while for 4 patients we used a 2.0 mm × 2.0 mm × 2.5 mm. The coarser grid was applied to match the resolution of vCT images which were resampled for these patients due to GPU memory constraints. We performed “per beam” and combined dose distribution analysis and calculated gamma pass rates with 2%/2 mm criterion using the standard-protocol CT volume as reference. 

## 3. Results

**Figure 6:**
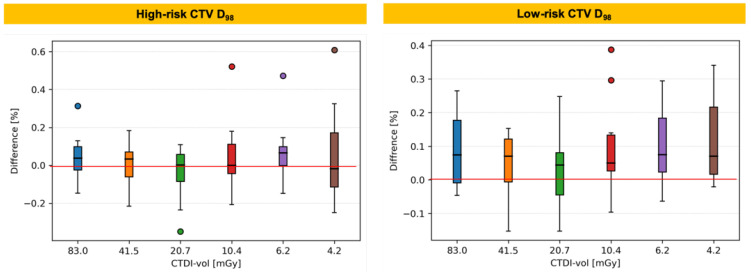
Percentage difference in target coverage (*D_98_*) between low-dose protocols and the reference standard protocol. Boxplots show: *median* (horizontal bar), *Q1–Q3 (25th–75th) percentile* (rectangle), *1.5 × (Q3–Q1) interquartile range* (whiskers), *outliers* (dots).

**Figure 7:**
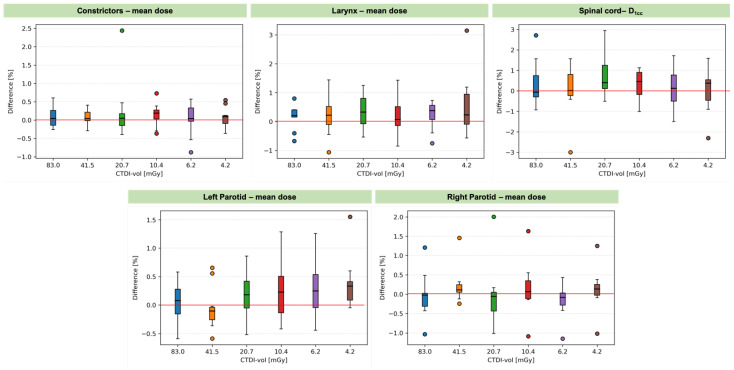
Percentage differences in OAR sparing metrics between low-dose protocols and the reference standard protocol. Boxplots show: *median* (horizontal bar), *Q1–Q3 (25th-75th) percentile* (rectangle), *1.5 × (Q3–Q1) interquartile range* (whiskers), *outliers* (dots).

Figure 6 shows the percentage difference in target coverage (*D_98_*) between low-dose protocols and the reference standard protocol. The differences as large as 0.6% for high-risk CTV and 0.4% for low-risk CTV are observed with no clear trend as the quality of CT image decreases. The difference in the median values did not exceed 0.1% and 0.2% for the high-risk and low-risk CTV, respectively. The corresponding percentage differences in OAR sparing metrics are shown in Figure 7. 

Differences of up to 3% in the mean dose (*D_mean_*) and *D_1cc_* (for the spinal cord) were observed. Similar to the target coverage, there is no trend as the imaging dose decreases. The difference in the median values of *D_mean_* was up to 0.5%. In the case of the spinal cord, the difference in the median values was below 1.7%. The metrics evaluated in the whole patient cohort for all the regions of interest are presented in Table 2. For all the studied scanning protocols, the median values satisfy the clinical goals. For almost all protocols, minimum *D*_98_ values were slightly below the clinical goal of 95%. This is due to considering only one fraction with the largest anatomical changes. For the parotid glands and constrictors, maximum *D_mean_* values vastly exceeded the dose corresponding to the clinical goal independent of the scanning protocol. This is because no constraint was applied to parotid glands (two patients) and to constrictors (one patient) in the treatment plan optimization due to the proximity of those organs to the target. 

**Figure 8:**
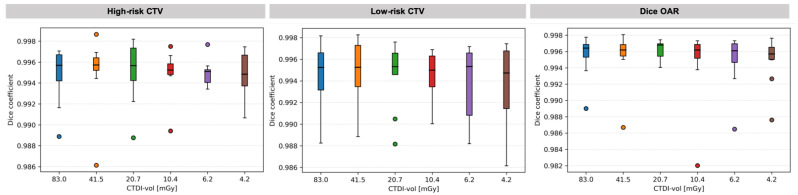
Similarity between contours propagated to low-dose CT and to the reference standard-protocol CT. Boxplots show: *median* (horizontal bar), *Q1–Q3 (25th–75th) percentile* (rectangle), *1.5 × (Q3–Q1) interquartile range* (whiskers), *outliers* (dots).

**Figure 9:**
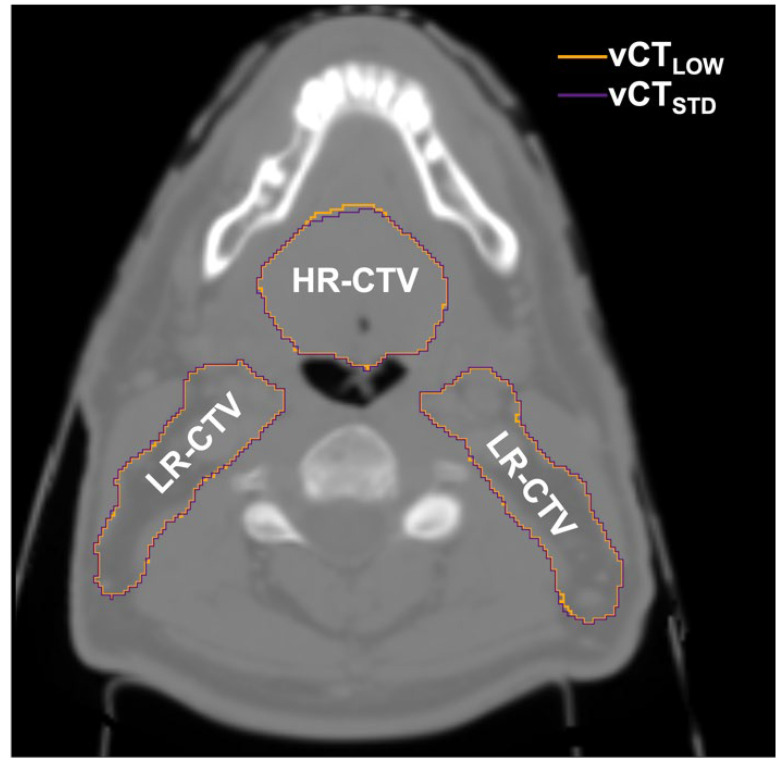
Difference between contours propagated to the standard-protocol CT (vCT_STD_) and the lowest-dose CT (vCT_LOW_) for the high-risk CTV (HR-CTV) and low-risk CTV (LR-CTV).

The similarity between contours propagated to low-dose CT and to the reference standard-protocol CT, quantified by the Dice coefficient, is shown in Figure 8 for all low-dose protocols. The Dice coefficient was found to be larger than 0.982 for all the contours in the whole patient cohort, while median values were all above 0.995 for all the protocols studied. As such, no significant influence of image quality on DIR was found. As in the case of DVH metrics, there was also no trend observed with the decrease in image quality. Figure 9 shows the magnitude of the differences between the contours for the target (high-risk and low-risk CTVs). The presented example corresponds to the lowest-dose protocol for a representative patient.

**Figure 10:**
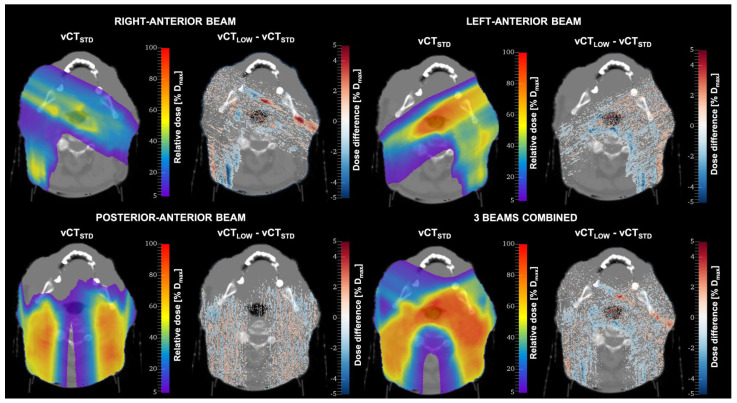
Dose distributions calculated for one patient in standard-protocol CT images (vCT_STD_) and the difference between the dose distributions calculated in vCT_STD_ and low-dose CT (vCT_LOW_).

Dose differences between the standard-protocol CT (vCT_STD_) and low-dose CT (vCT_LOW_) for one patient are shown in Figure 10. The vCT_LOW_ corresponds to the protocol with the lowest achievable imaging dose (CTDI*_vol_* = 4.2 mGy). Although for single voxels differences of up to 16% of the maximum dose (*D_max_*) were observed, the vast majority of voxels were within 5% *D_max_*. The results of 2%/2 mm gamma evaluation pass rates for the whole patient cohort are shown in Table 3. The results show the lack of uncertainties in proton range. This can be explained by the fact that the HU variations may cancel out over the beam path.

## 4. Discussion

This study focuses on the possibility of employing low-dose CT scanning protocols in clinical implementations of adaptive proton treatments. The main concern is how decreased image quality would affect contour propagation and dose calculation with respect to the standard protocol defined in the clinic for treatment planning. Our study represents the first attempt to quantify the influence of lowering the CT imaging dose on adaptation. We included several protocols in the study, defined by a gradual decrease in the tube current down to the minimum achievable in our CT scanner. 

The results clearly indicate that all the protocols studied lead to comparable dosimetric adaptation efficacy. The observed differences in DVH metrics with respect to the standard protocol were minimal and did not show any explicit dependency on the decreased image quality. In the analysis of the protocols, we also broke down the overall difference in adaptation into contour propagation and dose calculation. The contours obtained by deformable image registration were almost identical for all the protocols studied (Dice score above 0.98). The choice of the DIR algorithm is likely the source of much larger geometric uncertainty [33,34]. For the dose calculation, the protocol associated with the lowest imaging dose resulted in 2%/2 mm gamma pass rates over 99.95% for three beams and all ten patients, using the standard-protocol CT volume as reference. This confirms our expectation that the local differences due to the Gaussian noise in low-dose CT would average out and would not significantly influence the resulting dose distribution. The finding of the group from KU Leuven is consistent with our results on dose calculation accuracy [18]. They showed for three patient cases and one head phantom case that CT imaging dose reduction up to 90% does not have a significant effect on proton dose calculation. It should be noted that the CTDI*_vol_* values corresponding to the standard dose CT reported in that study were much lower than for our standard protocol. Therefore, for three of the four cases studied in that paper, the reduction in the imaging dose by 90% would give CTDI*_vol_* values similar to our lowest-dose protocol, which represents only 2.5% of our standard-protocol imaging dose. 

An alternative approach to dose calculation in a workflow based on low-dose CT would also be possible. The dose for each fraction could be calculated in the planning CT deformed with the vector field obtained from the DIR of the low-dose CT of the day to the planning CT. This method would be limited to DIR accuracy only, while the dose would be calculated in the high-quality CT image. However, there is a concern that the vector field would not capture the actual patient deformation and thus introduce artifacts.

Our study has certain limitations. The main limitation is the difficulty of generalizing the results to other CT scanners. Depending on the image reconstruction kernel and reconstruction algorithm, the noise can be lower for the same value of CTDI. For instance, iterative reconstruction would allow for a further decrease in the dose while keeping decent image quality [35]. As such, our study does not define universal CT scanning protocols for adaptive proton treatments. Instead, we show that a significant reduction in dose, in our case, a factor of 40, can be achieved without affecting the adaptation efficacy. Therefore, facilities planning to implement adaptive workflows based on CT imaging modality might consider using low-dose protocols after carefully evaluating the specific scanner and workflow. Another limitation of the study is the minimum tube current we could use in our scanner. Likely, an image with a much lower CTDI*_vol_* would still be suitable for adaptation. However, since we used a clinical scanner, there is a minimum setting that manufacturers impose to ensure the stability of CT numbers. Thus, the study could be extended by operating a CT scanner in a service/experimental mode. The definition of the noise in our study is relative to the standard-protocol CT image, which is assumed to be noise-free. In reality, even the high-dose CT has some level of noise, and two consecutively taken images would not be identical. We also limited our study to one fraction per patient. Given the fact the selected fraction corresponded to the largest changes in the patient’s geometry and the differences were insignificant, processing all the fractions would not affect the conclusions of the study.

One of the findings presented in this paper is that DIR performs well even in a significantly reduced quality image for head-and-neck cases. Other tumor sites, such as the abdomen, might require a better-quality CT, and it is likely that our lowest-dose protocol would lead to higher uncertainties.

In conclusion, low-dose CT is an attractive solution to limit the extra dose to the patient when performing daily adaptive proton therapy. Even the lowest achievable imaging dose in our scanner, 40 times lower than for the standard scanning protocol, allowed for producing images suitable for adaptation without compromising dosimetric predictions.

## Figures and Tables

**Table 1:** Scanning parameters used for phantom measurements. The standard protocol (STD) is highlighted in bold.

Tube Current	CTDI*_vol_*	Tube Voltage	Pitch Factor	Slice Thickness
**400 mA (STD)**	**166 mGy**	**140 kV**	**0.6**	**2.5 mm**
200 mA	83 mGy	140 kV	0.6	2.5 mm
100 mA	41.5 mGy	140 kV	0.6	2.5 mm
50 mA	20.7 mGy	140 kV	0.6	2.5 mm
25 mA	10.4 mGy	140 kV	0.6	2.5 mm
15 mA	6.2 mGy	140 kV	0.6	2.5 mm
10 mA	4.2 mGy	140 kV	0.6	2.5 mm

**Table 2:** Median (min–max) values for DVH metrics evaluated for all regions of interest (ROI) and studied protocols.

ROI	DVH Metric	CTDI*_vol_* (mGy)						
		166.0	83.0	41.5	20.7	10.4	6.2	4.2
High-risk CTV	*D*_98_ (%)	**97.1** (94.5–98.5)	**97.1** (94.8–98.6)	**97.1** (94.6–98.5)	**97.1** (94.6–98.5)	**97.2** (94.9–98.5)	**97.2** (95.0–98.5)	**97.2** (94.7–98.5)
Low-risk CTV	*D*_98_ (%)	**97.3** (95.5–98.3)	**97.4** (95.5–98.5)	**97.4** (95.6–98.4)	**97.4** (95.6–98.3)	**97.5** (95.6–98.6)	**97.4** (95.6–98.6)	**97.5** (95.5–98.6)
Constrictors	*D_mean_* (Gy)	**29.7** (7.0–61.2)	**29.7** (7.0–61.3)	**29.8** (7.0–61.2)	**29.7** (7.0–61.2)	**29.7** (7.0–61.3)	**29.8** (6.9–61.3)	**29.8** (7.0–61.1)
Right parotid	*D_mean_* (Gy)	**19.2** (12.5–55.4)	**19.2** (12.4–55.4)	**19.2** (12.5–55.3)	**19.2** (12.5–55.3)	**19.2** (12.5–55.4)	**19.1** (12.5–55.4)	**19.2** (12.5–55.4)
Left parotid	*D_mean_* (Gy)	**17.0** (9.9–52.4)	**17.0** (9.9–52.2)	**17.0** (10.0–52.4)	**17.0** (10.0–52.4)	**17.0** (10.0–52.5)	**17.0** (10.0–52.5)	**17.0** (10.0–52.4)
Larynx	*D_mean_* (Gy)	**20.5** (6.3–34.8)	**20.6** (6.4–34.6)	**20.5** (6.4–34.6)	**20.5** (6.4–34.6)	**20.4** (6.4–34.6)	**20.4** (6.4–34.5)	**20.5** (6.5–34.6)
Spinal cord	*D_1cc_* (Gy)	**12.1** (8.7–23.2)	**12.4** (8.6–23.2)	**12.3** (8.4–23.2)	**12.2** (8.9–23.2)	**12.2** (8.6–23.1)	**12.2** (8.6–23.1)	**12.2** (8.5–23.1)

**Table 3:** Mean and min–max 2%/2 mm gamma pass rates calculated in the low-dose CT volumes (CTDI*_vol_* = 4.2 mGy) using the standard-protocol CT volume as reference.

Beam	Mean (%)	Min–Max (%)
Posterior-Anterior	99.99	99.96–100.00
Left-Anterior	99.98	99.94–100.00
Right-Anterior	99.98	99.93–100.00
Combined	99.99	99.96–100.00

## Data Availability

The data are not publicly available due to privacy restrictions.
